# *Trichinella spiralis* Paramyosin Binds Human Complement C1q and Inhibits Classical Complement Activation

**DOI:** 10.1371/journal.pntd.0004310

**Published:** 2015-12-31

**Authors:** Ran Sun, Xi Zhao, Zixia Wang, Jing Yang, Limei Zhao, Bin Zhan, Xinping Zhu

**Affiliations:** 1 Department of Parasitology, School of Basic Medical Sciences, Capital Medical University, Beijing, PR China; 2 Department of Pediatrics, National School of Tropical Medicine, Baylor College of Medicine, Houston, Texas, United States of America; Instituto Butantan, BRAZIL

## Abstract

**Background:**

*Trichinella spiralis* expresses paramyosin (*T*s-Pmy) as a defense mechanism. *Ts*-Pmy is a functional protein with binding activity to human complement C8 and C9 and thus plays a role in evading the attack of the host’s immune system. In the present study, the binding activity of *T*s-Pmy to human complement C1q and its ability to inhibit classical complement activation were investigated.

**Methods and Findings:**

The binding of recombinant and natural *T*s-Pmy to human C1q were determined by ELISA, Far Western blotting and immunoprecipitation, respectively. Binding of recombinant *Ts*-Pmy (r*Ts*-Pmy) to C1q inhibited C1q binding to IgM and consequently inhibited C3 deposition. The lysis of antibody-sensitized erythrocytes (EAs) elicited by the classical complement pathway was also inhibited in the presence of r*T*s-Pmy. In addition to inhibiting classical complement activation, r*Ts*-Pmy also suppressed C1q binding to THP-1-derived macrophages, thereby reducing C1q-induced macrophages migration.

**Conclusion:**

Our results suggest that *T*. *spiralis* paramyosin plays an important role in immune evasion by interfering with complement activation through binding to C1q in addition to C8 and C9.

## Author Summary

Trichinellosis is one of the most important food-borne parasitic zoonoses worldwide. The key factor for *Trichinella spiralis* to survive in its host is evading from the attacks by the immune defense system. Our previous study revealed that paramyosin from *Trichinella spiralis* (*Ts*-Pmy) played a role in evading host immune attacks by binding to human complement C8 and C9. Here, we demonstrated that *Ts*-Pmy inhibited classical complement activation by binding to human complement C1q. As a result, classical complement pathway-mediated hemolysis was inhibited in the presence of *Ts*-Pmy. Additionally, *Ts*-Pmy inhibited C1q binding to THP-1-derived macrophages and C1q-induced macrophages migration. These results suggest that *Trichinella spiralis* paramyosin is a potential immunomodulator involved in the evasion of the host complement attack by binding to C1q in addition to C8/C9, and therefore is a potent vaccine target against trichinellosis.

## Introduction

Trichinellosis is a serious zoonotic disease caused by the ingestion of undercooked meat contaminated with the larvae of *Trichinella spiralis*. Heavy infection can result in death [1]. Recently, trichinellosis has been regarded as an emerging or re-emerging disease in some countries due to improvements in people’s living standards and changes in eating habits [2,3]. To establish parasitism in the host, *T*. *spiralis* has evolved sophisticated mechanisms to avoid immune attack from the host. Elucidating the mechanisms developed by the parasite to survive in the host would facilitate the development of strategies to interrupt parasitism and prevent infection.

The complement system is considered to be the first line of defense against invaded pathogens and plays a crucial role in human innate immunity [4]. Many pathogens have evolved diverse strategies to evade host immune attacks and that commonly encounter the complement system first. The human astrovirus coat protein inhibited classical and lectin pathway activation by binding to C1q and mannan binding lectin (MBL) [5,6]. Other pathogenic proteins,such as *Pseudomonas aeruginosa* alkaline protease and *Trypanosoma carassii* calreticulin, also interfere with complement activation by binding to complement components [7,8]. Many parasitic helminths release molecules that interfere with the functions of complement and assist in the parasite’s survival in the host [9,10]. One protein that has been well studied for its immunomodulatory effect on the host complement system is paramyosin [11–13]. Paramyosin is a protein dimer that forms thick myofilaments and found exclusively in invertebrates [14]. Recent studies on paramyosin suggested that it was a functional protein involved in helminth infection as well as a structural protein [12–16]. Many helminth parasmyosins have been reported to be capable of directly reacting with human complement C8 or/and C9. *Schistosoma mansoni* paramyosin protected the parasites against host attack by binding to complement C8 and C9 [12,15]. *Clonorchis sinensis* paramyosin bound both human collagen and C9 [16]. In our previous study, we have identified that *T*. *spiralis* paramyosin (*Ts*-Pmy) was expressed on the surface of *T*. *spiralis* adult and larval worms [13]. Mice immunized with recombinant *Ts*-Pmy (r*Ts*-Pmy) achieved protective immunity against *T*. *spiralis* infection [17], suggesting that it was a good vaccine candidate. Further investigations into its role in the survival of parasites in the host demonstrated its inhibitory effect on the formation of the complement membrane attack complex (MAC) by interacting with complement C8 and C9. As a consequence, the invaded *T*. *spiralis* could evade the host complement attack by inhibiting MAC formation [13,18]. The C9 binding site on *Ts*-Pmy was determined to be located within the C-terminus of the protein (between ^866^Val and ^879^Met) [18]. A monoclonal antibody (mAb 9G3) targeting the binding site could block the binding of *Ts*-Pmy to human C9, resulting in a significant increase in the complement-mediated killing of newborn larvae of the parasite *in vitro* [18,19].

In addition to targeting C8 and C9 by helminth-expressed paramyosin, it was reported that *S*. *mansoni* and *Taenia solium* produced paramyosin proteins could bind to complement C1q [11]. C1q is the first complement component and initiates the classical activation pathway. To determine whether *Ts*-Pmy also inhibited classical complement pathway through binding to C1q, except for C8/C9, as a sophisticated strategy to evade host complement attack, the interaction between *Ts*-Pmy and complement C1q was investigated. In this study, we demonstrated that r*Ts*-Pmy was able to bind to C1q indeed, resulting in the inhibition of classical complement activation. Thus, C1q represented a complement component and pathway targeted by *Ts*-Pmy in addition to C8 and C9 as a strategy to escape host immune response.

## Materials and Methods

### Animals

Female BALB/c mice aged 6–8 weeks and free of specific pathogens were obtained from the Laboratory Animal Services Center of Capital Medical University (Beijing, China). The mice were maintained under specific pathogen-free condition with suitable humidity and temperature. All experimental procedures were approved by the Capital Medical University Animal Care and Use Committee (approval number: 2012-X-108) and comply with the NIH Guidelines for the Care and Use of Laboratory Animals.

### Sera

Normal human serum (NHS) was derived from the blood of 20 healthy human volunteers, aliquoted and frozen at −80°C. All human blood samples were collected according to the protocol approved by the Institutional Review Board (IRB) of Capital Medical University. Human C1q-deficient serum (C1q D) and C3-deficient serum (C3 D) were purchased from Merck (Germany).

### Parasites, antigens, antibody and recombinant protein preparations

*T*. *spiralis* (ISS533) was maintained in female ICR mice. Muscle larvae (ML) were recovered from infected mice using a modified pepsin-hydrochloric acid digestion method as previously described [20]. Adult worms were collected from the intestines of infected mice four days following oral larval challenge. Crude adult worm antigens were prepared from homogenized worm extracts based on a previously described protocol [18]. The anti-*Ts*-Pmy monoclonal antibody (mAb) 9G3 that specifically recognized *Ts*-Pmy was previously produced [19]. Recombinant *T*s-Pmy (r*Ts*-Pmy) with a His-tag at the C-terminus was expressed in baculovirus/insect cells (Invitrogen, USA) and purified by Ni-affinity chromatography (Qiagen, USA). *T*s87 (38 kDa) was an excretory-secretory antigen of *T*. *spiralis* identified previously [21]. In this study, recombinant *Ts*87 (r*T*s87) was used as a non-relevant protein control.

### Cell culture

The human leukemia monocytic cell line THP-1 was purchased from China Infrastructure of Cell Line Resource. THP-1 cells were induced into M0 phenotype macrophages by incubating with phorbol-12-myristate-13-acetate (PMA, Sigma, USA) for 48 h and M2 by stimulating with human IL-4 (PeproTech, USA) in RPMI 1640 medium containing 10% FBS at 37°C in 5% CO_2_ for another 24 h [22].

### Binding of *T*s-Pmy to human complement C1q

#### ELISA

To determine whether r*Ts*-Pmy bound to complement C1q, plates were coated with different amounts of human C1q (0, 0.5, 1.0, 1.5 μg) and BSA (2 μg) in 100 μl of coating buffer (100 mM Na2CO3/NaHCO3, pH 9.6) at 4°C overnight. After washing three times with 1× phosphate buffered saline (PBS) pH 7.4 containing 0.05% Tween-20 (PBST), the plates were blocked with PBS containing 2% BSA for 2 h at 37°C. The different amounts of r*Ts*-Pmy (0, 1, 2, 3, 4 μg) in 100 μl of 20 mM Tris-HCl, 50 mM NaCl and 1 mM CaCl2, pH 7.4 were added for 1 h at 37°C. The plates were washed three times with PBST, the binding of r*Ts*-Pmy to human C1q was determined with anti-*Ts*-Pmy monoclonal antibody 9G3 (1:2,500 in 1% BSA/PBS). HRP-conjugated goat anti-mouse IgG (BD Biosciences, USA; 1:10,000 in 1% BSA/PBS) was used as the secondary antibody and o-phenylendiamine dihydrochloride (OPD, Sigma, USA) was used as the substrate. The absorbance of the supernatant was measured at 450 nm with a MultiskanGO plate reader (Thermo, USA).

#### Far Western blotting

To further demonstrate the interaction between r*Ts*-Pmy and complement C1q, purified human C1q (5 μg; Merck, Germany) under reducing condition were subjected to SDS-PAGE using 12% polyacrylamide gel followed by either Coomassie bule staining or Far Western blotting. After blocking with 5% dry milk in 1 × PBS, the membrane was incubated with r*Ts*-Pmy (5 μg/ml in PBST and 1% dry milk) at 37°C for 2 h and then washed with PBST. The membrane was probed with an anti-His mAb (r*Ts*-Pmy contains His-tag) (Tiangen, China; 1:5,000 in PBST containing 1% dry milk) for 1 h at room temperature. IRDye 800 CW-labeled goat anti-mouse IgG (LI-COR, Germany; 1:10,000 in PBST containing 1% dry milk) was used as the secondary antibody. Vice versa, r*Ts*-Pmy, non-relevant control BSA and r*Ts*87 (5 μg each) were subjected to 12% SDS-PAGE and then transferred to a NC membrane. After blocking, the membrane was probed with human complement C1q (5 μg/ml in PBST containing 1% dry milk) at 37°C for 2 h. The anti-C1q mAb (Abcam, USA; 1:1,000 in PBST containing 1% dry milk) was used as the primary antibody and IRDye 800 CW-labeled goat anti-mouse IgG (1:10,000 in PBST containing 1% dry milk) was used as the secondary antibody. Membranes were visualized and imaged using an Odyssey infrared imaging system (LI-COR, Germany).

#### Immunoprecipitation

To verify whether native *Ts*-Pmy from *T*. *spiralis* was able to bind to C1q, Protein G Micro Beads (Miltenyi Biotec, Germany) were pre-incubated with the anti-*Ts*-Pmy mAb 9G3 (2 μg) and *T*. *spiralis* adult worm crude extracts (40 μg) for 30 min on ice. Then, human complement C1q (3 μg) was added and the incubation was continued for 2 h at 4°C. The beads were washed four times with washing buffer (1% NP40 substitute, 50 mM Tris buffer, pH 8.0), and the bound proteins were eluted in 1× SDS gel loading buffer by boiling for 5 min. The eluted proteins were subjected to 12% SDS-PAGE and transferred to a NC membrane. The membrane was probed with an anti-C1q mAb (Abcam, USA) at a 1:1,000 dilution in PBST containing 1% dry milk; IRDye 800CW-labeled goat anti-mouse IgG (1:10,000 in PBST containing 1% dry milk) was used as the secondary antibody.

### C3 deposition assay

To evaluate whether the binding of r*Ts*-Pmy to C1q inhibited complement activation, C3 deposition following complement activation were analyzed [23]. Plates were coated with 2 μg/ml of human IgM in 100 μl of coating buffer (100 mM Na2CO3/NaHCO3, pH 9.6) at 4°C overnight. After washing three times with PBST, the plates were blocked with 1 × PBS containing 5% BSA for 2 h at 37°C. Two μg of C1q was pre-incubated with different amounts of r*Ts*-Pmy (0, 2, 4 μg) and BSA (4 μg, as a control) for 2 h at 37°C before adding to the plates coated with IgM (the activator) for 1 h at 37°C. After washing three times with PBST, C1q-deficient serum (C1q D) diluted to 2% in GVBS^++^ (Veronal-buffered saline containing 1 mM MgCl2, 0.15 mM CaCl2, 0.05% Tween-20, and 0.1% gelatin, pH 7.4) was added as a source of rest complement components to the plates for 1 h at 37°C and then washed with PBST three times. C3 deposition was determined with anti-C3 polyclonal antibody (Abcam, USA; 1:5,000 in 1% BSA/PBS). HRP-conjugated goat anti-rabbit IgG (BD Biosciences, USA) was used as the secondary antibody and OPD (Sigma, USA) was used as the substrate. The absorbance of the supernatants was measured at 450 nm with a MultiskanGO plate reader (Thermo, USA).

### Hemolytic assay

To determine the inhibition of classical complement activation-mediated hemolysis by r*Ts*-Pmy, freshly prepared sheep red blood cells (RBC) (10^9^ cells/ml) were sensitized with an anti-sheep RBC antibody (Sigma, USA) at a 1:200 dilution in 1× HBSS^++^ (Hank’s balanced salt solution containing 1 mM MgCl_2_, 0.15 mM CaCl_2_. Thermo, USA) at 37°C for 30 min, then washed with 1× HBSS^++^. Different amounts of r*T*s-Pmy (0, 1, 2, 4 μg) were pre-incubated with NHS (5% in 1× HBSS^++^) for 1 h at 37°C and then added to the antibody-sensitized erythrocytes (EAs) (5×10^7^ cells/well) for 30 min at 37°C. Cold HBSS^++^ containing 10 mM EDTA was added to stop the reaction. The cells were centrifuged at 3,000 rpm for 10 min. The absorbance of the supernatants was measured at 412 nm with a MultiskanGO plate reader (Thermo, USA). The percent lysis was calculated relative to cells completely lysed in water.

### Inhibtion of r*Ts*-Pmy on the binding of C1q to IgM

To determine the inhibition of r*Ts*-Pmy on the binding of C1q to IgM, the ELISA assay was performed. Plates were coated with 2 μg/ml of human IgM in 100 μl of coating buffer (100 mM Na2CO3/NaHCO3, pH 9.6) at 4°C overnight. After washing three times with PBST, the plates were blocked with 1 × PBS containing 2% BSA for 2 h at 37°C. One μg of C1q was pre-incubated with different amounts of r*Ts*-Pmy or BSA (0, 2, 3, 4 μg) for 2 h at 37°C, then added to the plates coated with IgM for 1 h at 37°C. After washing three times with PBST, the binding of C1q to IgM was determined with anti-C1q polyclonal antibody (Abcam, USA; 1:3,000 in 1% BSA/PBS). HRP-conjugated goat anti-rabbit IgG (BD Biosciences, USA) was used as the secondary antibody and OPD (Sigma, USA) was used as the substrate. The absorbance of the supernatants was measured at 450 nm with a MultiskanGO plate reader (Thermo, USA).

### Cell immunofluorescence labeling

To evaluate whether r*Ts*-Pmy could inhibit C1q binding to macrophages, THP-1 cells (containing C1q receptors, 2×10^5^ cells/ml) [24,25] were induced into M2 type macrophages with PMA and human IL-4 as previously described [22] because M2 phenotype macrophages play a role in the immune response to helminth infections [26]. The cells were fixed with 4% paraformaldehyde (PFA) for 20 min at room temperature and then washed with PBS. The cells were blocked with goat serum (ZSGB-BIO, China) for 30 min at room temperature before adding C1q (80 μg/ml) that was pre-incubated with r*T*s-Pmy (80 μg/ml). The incubation was continued at 37°C for 1 h. After washing with PBS, rat anti-C1q mAb (Abcam, USA; 1:100 in PBS) was added; Dylight 488-labeled goat anti-rat IgG (KPL, USA; 1:100 in PBS) was used as the secondary antibody. The control group incubated with r*Ts*-Pmy was detected by anti-*Ts*-Pmy antibody 9G3. The cell nuclei were stained with DAPI (ZSGB-BIO, China). Images were acquired with an inverted fluorescence microscope (Leica, DM4000B), and the fluorescence intensity of C1q binding to macrophages was measured with high content analysis (Thermo, USA).

### Chemotaxis assay

The effect of r*T*s-Pmy on the C1q-induced migration of THP-1-derived macrophages was determined using a Transwell insert with an 8 μm membrane (Corning, USA) [27]. A total of 1×10^7^ THP-1 cells were added into the upper chamber and stimulated with 100 nM PMA for 48 h; then, human IL-4 (20 nM) was added for another 24 h to induce into M2 type macrophages. Human C1q (10 nM) with different amounts of r*T*s-Pmy (0, 3, 6, or 12 μg) was added into the lower chamber. The incubation was continued at 37°C in 5% CO_2_ for 24 h to allow the cells to migrate through the membrane. After washing with PBS, the cells on the membrane were fixed with methanol and stained with Giemsa. The cells that remained in the upper surface of the membranes were removed, and the cells that migrated to the bottom surface of the membranes were counted using a phase contrast microscope (Leica, 1X71). A total of 8 randomly selected fields were counted, and the average of each field was calculated using a previously described method [28]. Non-relevant BSA (12 μg) was added as a negative control, and LPS (100 ng/ml) was used as a positive control.

### Statistical analysis

The data were expressed as the means ± standard deviations (S.D). Differences between groups were evaluated with the GraphPad Prism 5 software (San Diego, CA, USA) using one-way ANOVA. *p* < 0.05 was considered statistically significant.

## Results

### Binding of r*Ts*-Pmy to human complement C1q

**Fig 1:**
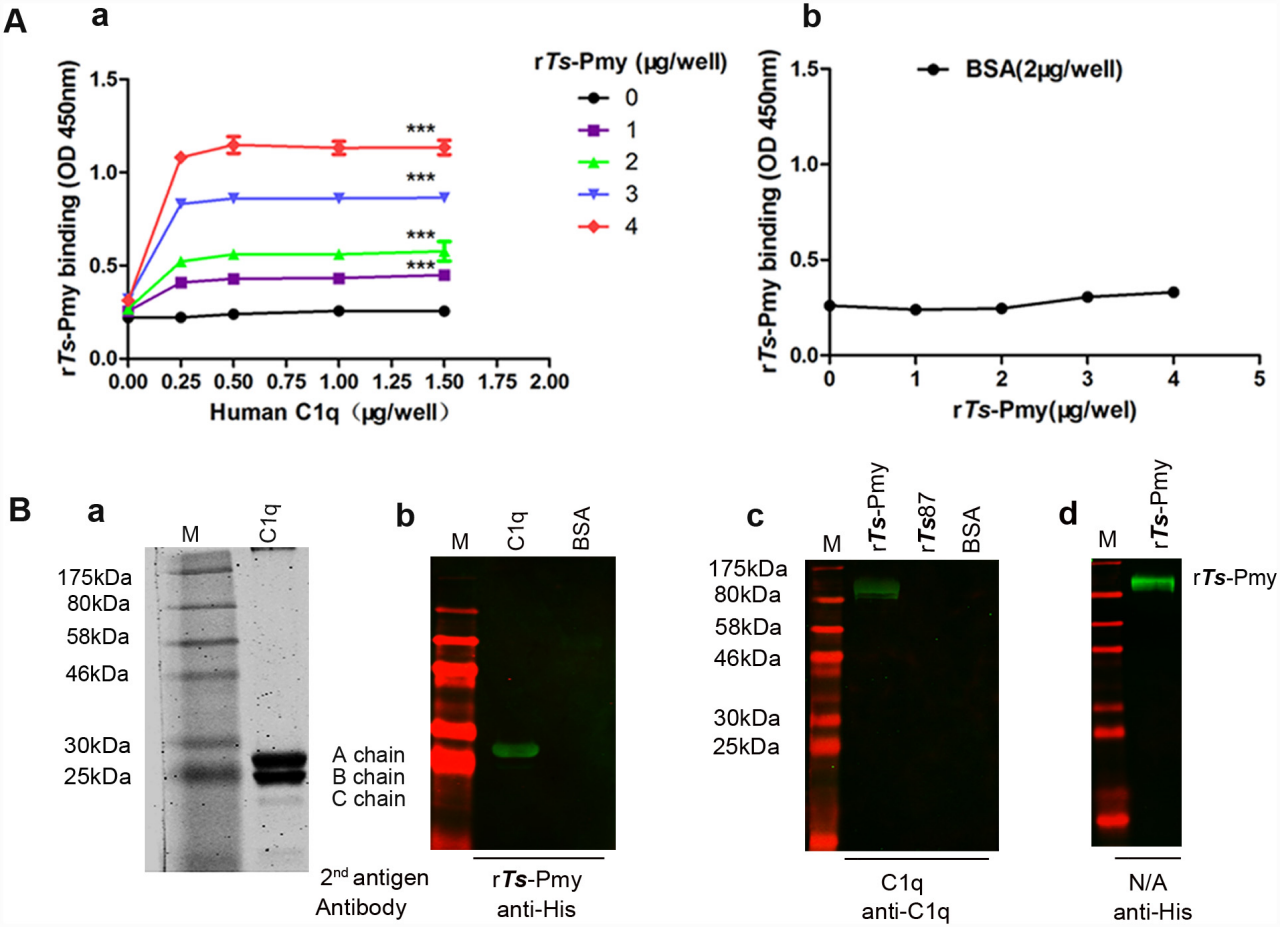
r*Ts*-Pmy bound to A chain of human C1q. **(A)** 96-well plates were coated with different amounts of C1q (a) or 2 μg BSA (b). Increasing amounts of r*Ts*-Pmy were added and anti-*Ts*-Pmy antibody 9G3 was used to detect the binding. ***p<0.001. **(B)** Human C1q (5 μg) was subjected to SDS-PAGE under reducing condition and stained with Coomassie blue (a). C1q and BSA (each 5 μg) under reducing condition were transferred onto a NC membrane, incubated with r*Ts*-Pmy (5 μg/ml) and detected with anti-His mAb (1:5,000) (b). The same amount of r*Ts*-Pmy, r*Ts*87 or BSA (each 5 μg) were transferred to a NC membrane and then incubated with C1q (5 μg/ml). The proteins bound on the membrane were detected with anti-C1q antibody (1:1,000) (c). The r*Ts*-Pmy on the membrane was detected with anti-His mAb (d). The consistent results are repeated for three times. M, standard protein marker.

The binding of r*Ts*-Pmy to human complement C1q was determined by ELISA and Far Western blotting. ELISA results clearly showed that r*Ts-*Pmy bound to human C1q coated plates in a dose dependent manner while BSA coated plates (2 μg/well) showed no any binding to r*Ts*-Pmy (Fig 1A). Wells coated with 0.5 μg of C1q had showed saturate binding with r*Ts*-Pmy. SDS-PAGE results showed that C1q was separated into 3 chains (A, B and C chain) under reducing condition (Fig 1Ba). Interestingly, Far Western blotting demonstrated that only A chain of C1q was bound to r*Ts*-Pmy, as detected by the anti-His antibody (r*Ts*-Pmy contain a 6His-tag at the C-terminus) (Fig 1Bb), no binding was observed to the non-relative control BSA. Vice versa, r*Ts*-Pmy under reducing condition was bound to C1q as detected by the anti-C1q antibody (Fig 1Bc). C1q did not bind to the same amount of r*Ts*87 or BSA. The results confirmed that r*Ts*-Pmy specifically bound to the A chain of human C1q.

### Binding of native *Ts*-Pmy from worms to human complement C1q

**Fig 2:**
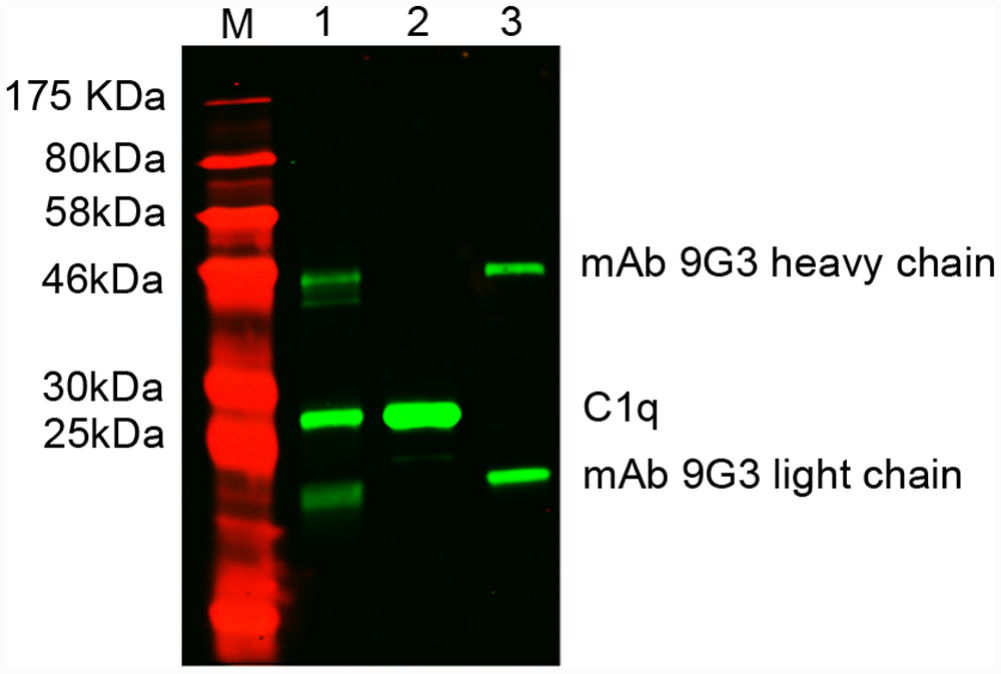
Native *T*s-Pmy from *T*. *spiralis* worm bound to human C1q. Protein G Micro Beads were pre-incubated with anti-*T*s-Pmy mAb 9G3 and *T*. *spiralis* adult worm crude extracts. Then, human complement C1q was added and the incubation was continued for 2h. The bound proteins were subjected to SDS-PAGE and transferred to a NC membrane. The membrane was probed with an anti-C1q mAb. The consistent results are repeated for three times. M, standard protein marker; Lane 1, pulled down immune complex (worm extracts + anti-*Ts*-Pmy + C1q); Lane 2, human C1q alone (2 ug); Lane 3, human C1q with the anti-*T*s-Pmy mAb 9G3 alone without the addition of *T*. *spiralis* adult extracts.

The binding of native *T*s-Pmy from *T*. *spiralis* adult worms to human C1q was investigated by immunoprecipitation and Western blotting (Fig 2). The results clearly demonstrated that C1q bound to native *Ts*-Pmy from worm extracts and that the binding complex was pulled down by the anti-*Ts*-Pmy mAb 9G3. No C1q was pulled down by the mAb 9G3 alone, indicating that C1q bound specifically to native *Ts*-Pmy.

### Inhibition of complement activation by r*T*s-Pmy

**Fig 3:**
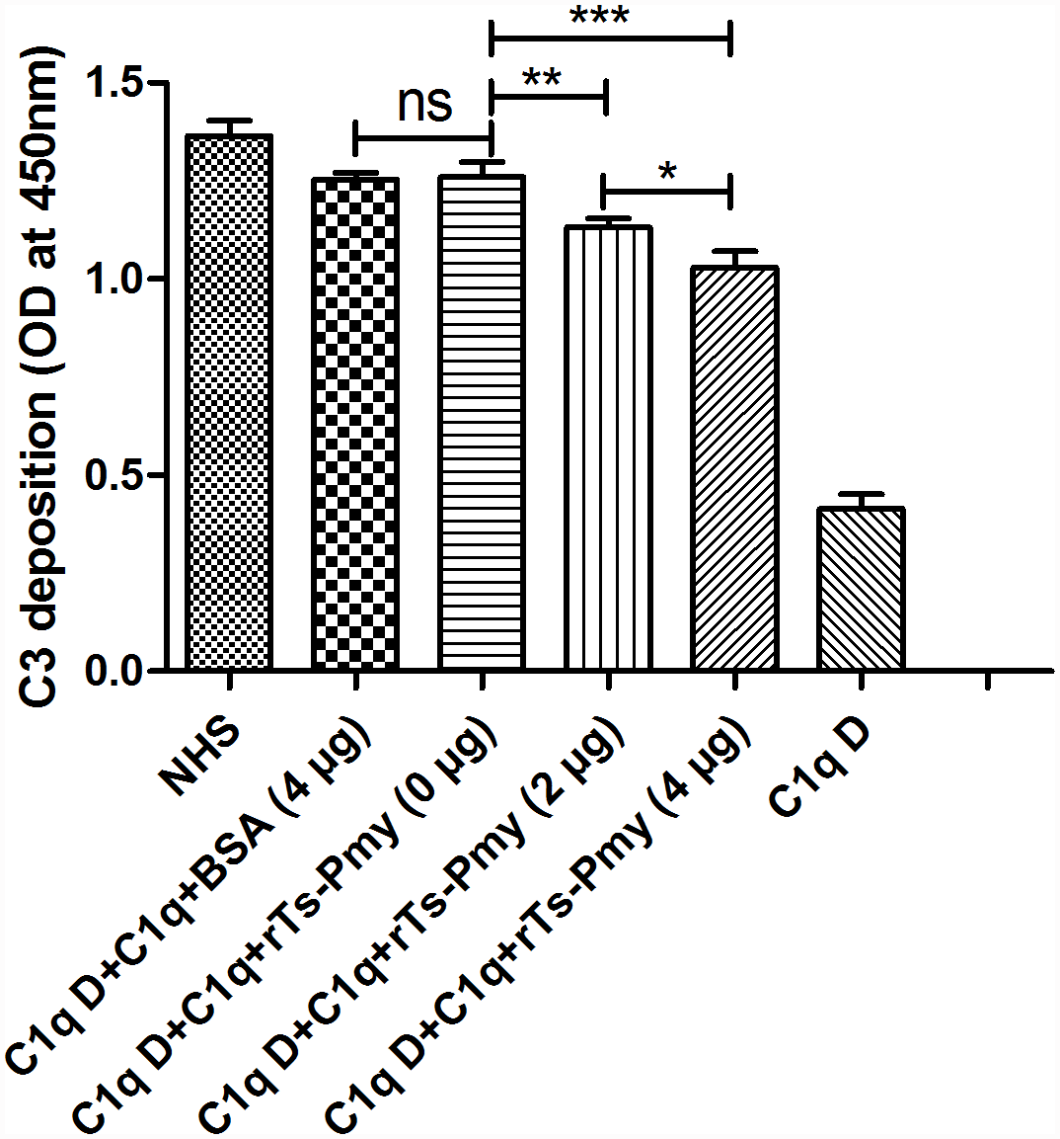
Classical complement activation was inhibited by r*Ts*-Pmy. Two μg of human C1q was pre-incubated with increasing amounts of r*T*s-Pmy (0, 2, 4 μg) or BSA (4 μg), then added to human IgM-coated plates. After washing with PBST for three times, a total of 2% C1q-deficient serum (C1q D) was added as a source of rest complement components. The deposition of C3 was detected with anti-C3 polyclonal antibody. The NHS alone and BSA added to the activation were used as controls. The results are shown as the means ± SD for three independent experiments.* *p*<0.05, **p<0.01, ***p<0.001. ns, no significant difference.

To evaluate whether the binding of r*Ts*-Pmy to C1q inhibits classical complement activation, we analyzed C3 deposition on plates coated with human IgM in the presence of different amounts of r*Ts*-Pmy. The result showed that activation of C1q deficient serum (C1q D) was able to be reconstituted with the addition of C1q to the similar level of NHS by detecting C3 deposition (C1q D+C1q). However, the addition of increasing amounts of r*Ts*-Pmy (0, 2, 4 μg) to C1q decreased C3 deposition in a dose dependent manner and the difference between the doses was significant (Fig 3). BSA (4 μg) had no any inhibitory effect with the C3 deposition similar to the group without any *Ts*-Pmy added (C1q D+C1q+*Ts*-Pmy 0 μg). C1q D itself didn’t cause significant C3 deposition without addition of C1q. The result demonstrated that activation of the classical complement pathway was inhibited by the binding of r*T*s-Pmy to C1q in this study.

### Inhibition of classical complement-mediated hemolysis by r*T*s-Pmy

**Fig 4:**
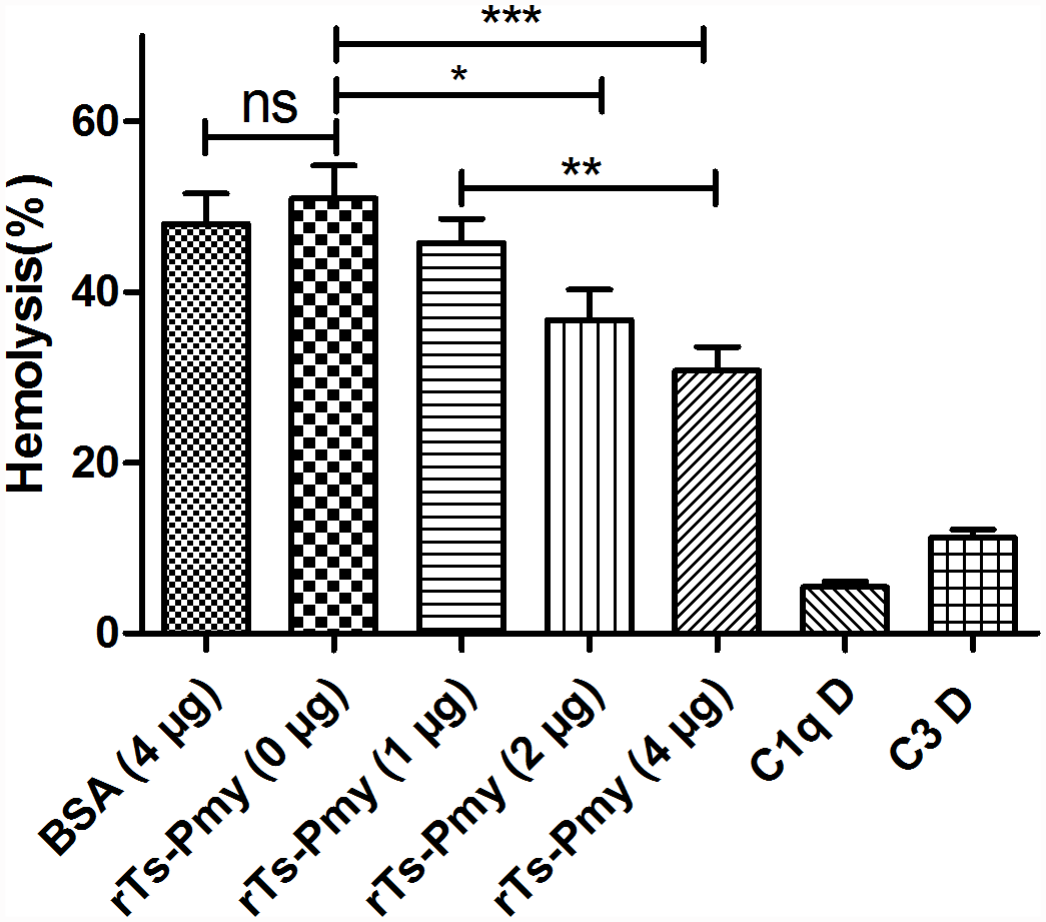
Complement-mediated hemolysis was inhibited by r*T*s-Pmy. Normal human serum (NHS) was pre-incubated with r*T*s-Pmy (0, 1, 2, or 4 μg) for 1 h prior to incubation with EAs. Hemolysis was measured at 412 nm for the supernatant of the reactions compared to complete lysis in water. The results shown are means ± SD and are representative of three independent experiments. BSA, C1q D and C3 D alone were added as controls. *p<0.05, **p<0.01, ***p<0.001. ns, no significant difference.

To further determine whether r*T*s-Pmy inhibited classical complement activation, antibody-sensitized sheep erythrocytes (EAs) were incubated with fresh NHS pre-incubated with different amounts of r*Ts*-Pmy. The classical complement-mediated hemolysis results showed that the lysis of EAs was significantly inhibited by the addition of r*Ts*-Pmy in a dose-dependent manner (Fig 4). There was no significant hemolysis in the presence of C1q D or C3 D serum (C1q- or C3-deficient) because the classical pathway could not be activated without C1q or C3. BSA had no inhibitory effect on classical complement activation.

### Inhibition of r*Ts*-Pmy on the binding of C1q to IgM

**Fig 5:**
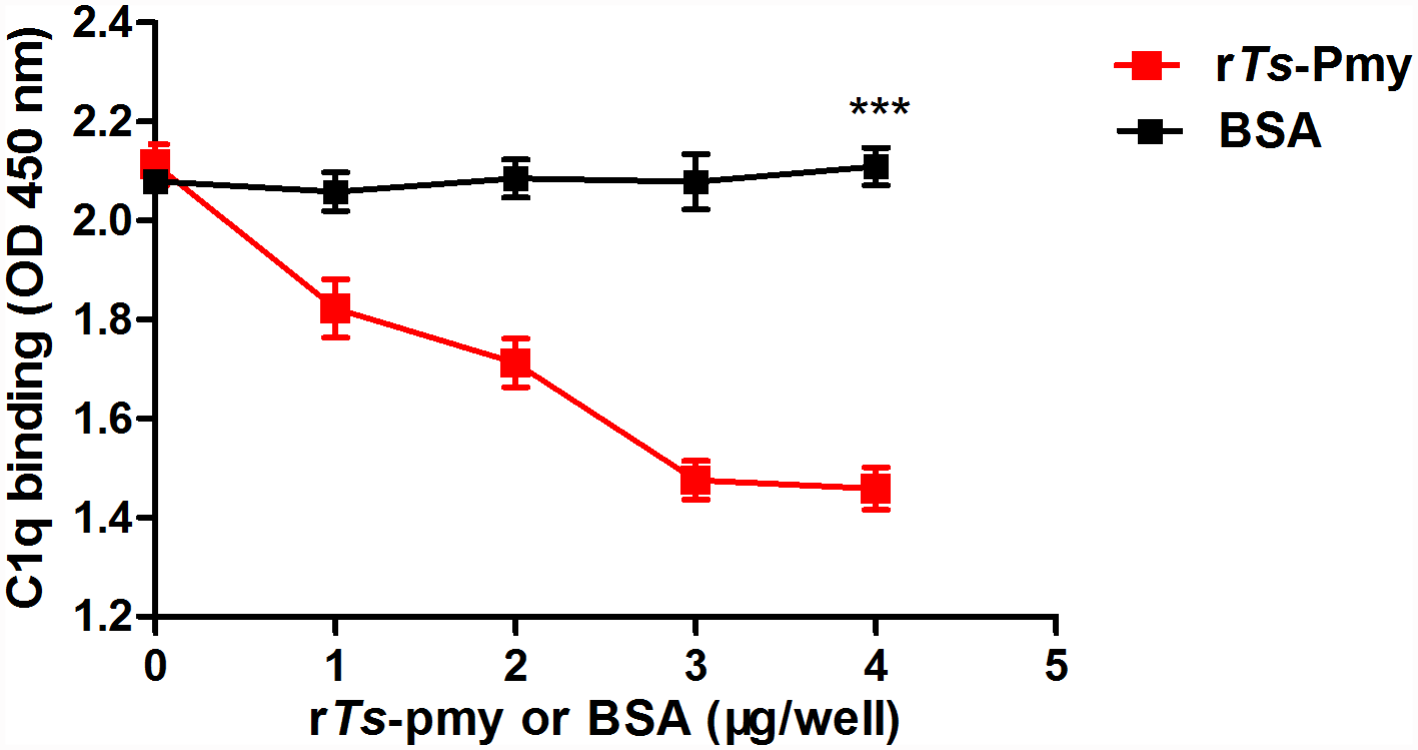
r*Ts*-Pmy inhibited the binding of human C1q to IgM. One μg of C1q was pre-incubated with different amounts of r*Ts*-Pmy or BSA (0, 2, 3, 4 μg), then added to the IgM coated plates. After washing three times with PBST, the binding of C1q to IgM was determined with anti-C1q polyclonal antibody. The results shown are means ± SD and are representative of three independent experiments. ***p<0.001.

In order to understand how r*Ts*-Pmy is involved in the inhibition of classical complement activation, different amounts of r*Ts*-Pmy were incubated with C1q before adding into IgM coated plate. It was reported that IgM bound to the head region of C1q [29]. Interestingly, our result demonstrated that the binding of human C1q to IgM was inhibited in the presence of r*Ts*-Pmy in a dose dependent manner. There was no inhibitory effect was observed when the same amount of BSA was added (Fig 5). This result implied that IgM and r*Ts*-Pmy bound to the same region of human C1q and pre-incubation with r*Ts*-Pmy blocked the region on C1q that binds to IgM, suggesting the binding site of r*Ts*-Pmy was on the head region of C1q.

### Inhibition of the binding of C1q THP-1-derived macrophages by r*T*s-Pmy

**Fig 6:**
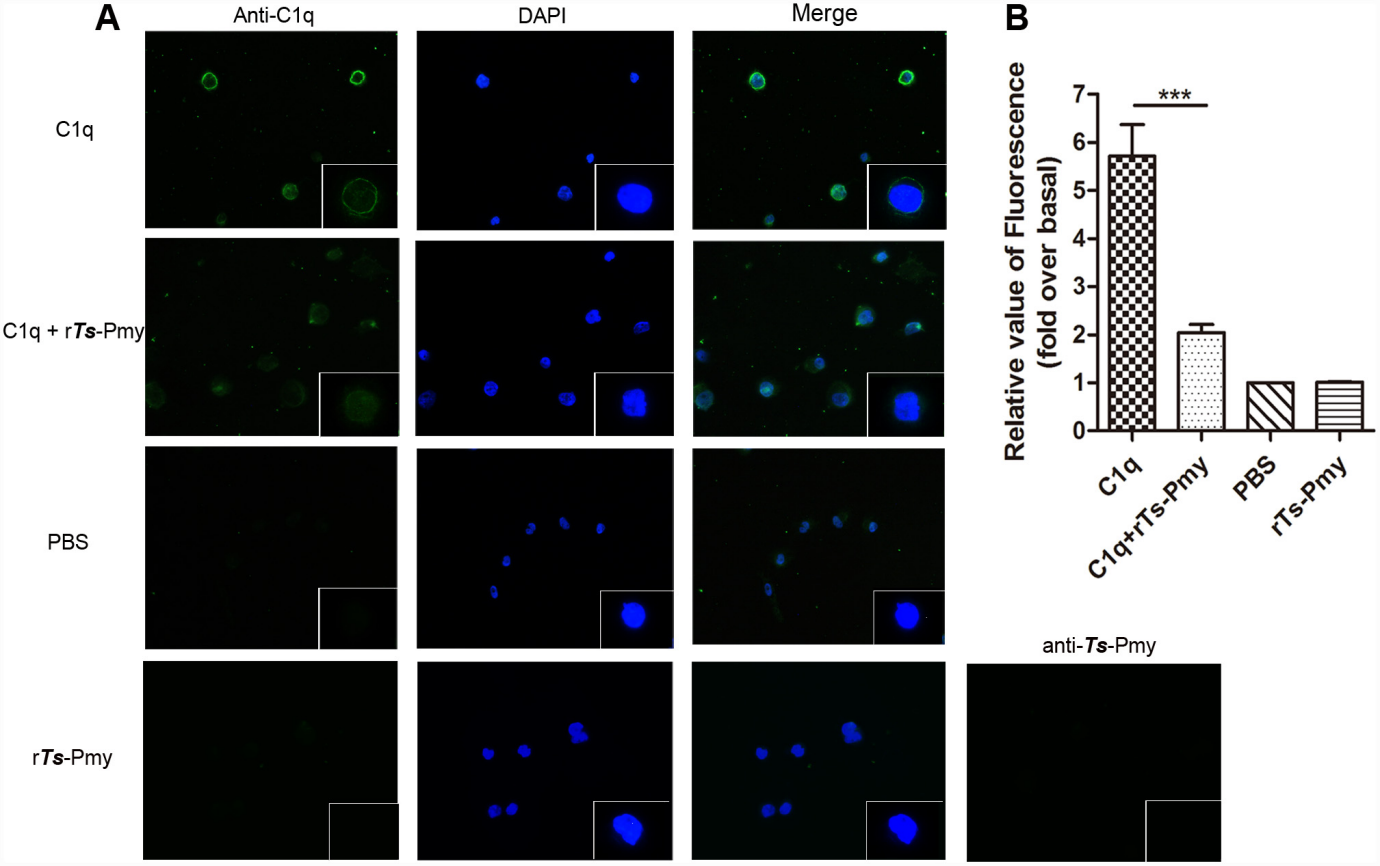
The binding of C1q to THP-1-derived macrophages was inhibited by r*T*s-Pmy. **(A)** After fixing with 4% paraformaldehyde and blocking with goat serum, M2 type macrophage cells were incubated with C1q, C1q plus r*T*s-Pmy or PBS alone for 1 h at 37°C, and then detected with an anti-C1q mAb or anti-*Ts*-Pmy mAb 9G3. Dylight 488 was used as a secondary antibody (green). The nuclei were stained with DAPI (blue). The imagine magnitude is 400 X with one cell 1000 X at the bottom right corner. **(B)** The fluorescence intensity was measured with high content analysis. The results shown are means ± SD and are representative of three independent experiments. ***p<0.001.

To assess whether r*Ts*-Pmy affected C1q binding to THP-1-derived macrophages [25,30], C1q was pre-incubated with r*T*s-Pmy before adding to THP-1-derived macrophages. Immunofluorescence staining with anti-C1q mAb showed that the fluorescence intensity on macrophage cells was decreased after C1q was incubated with r*Ts*-Pmy (C1q+r*T*s-Pmy) (Fig 6A). No fluorescence was detected in the PBS and r*Ts*-Pmy alone control group. The quantitative measurement showed that the fluorescence intensity was significantly decreased in C1q with r*T*s-Pmy group compared with C1q only group (Fig 6B). The results indicated that r*T*s-Pmy interfered with the binding of C1q to macrophages.

### Inhibition of C1q-induced chemotaxis of THP-1-derived macrophages by r*T*s-Pmy

**Fig 7:**
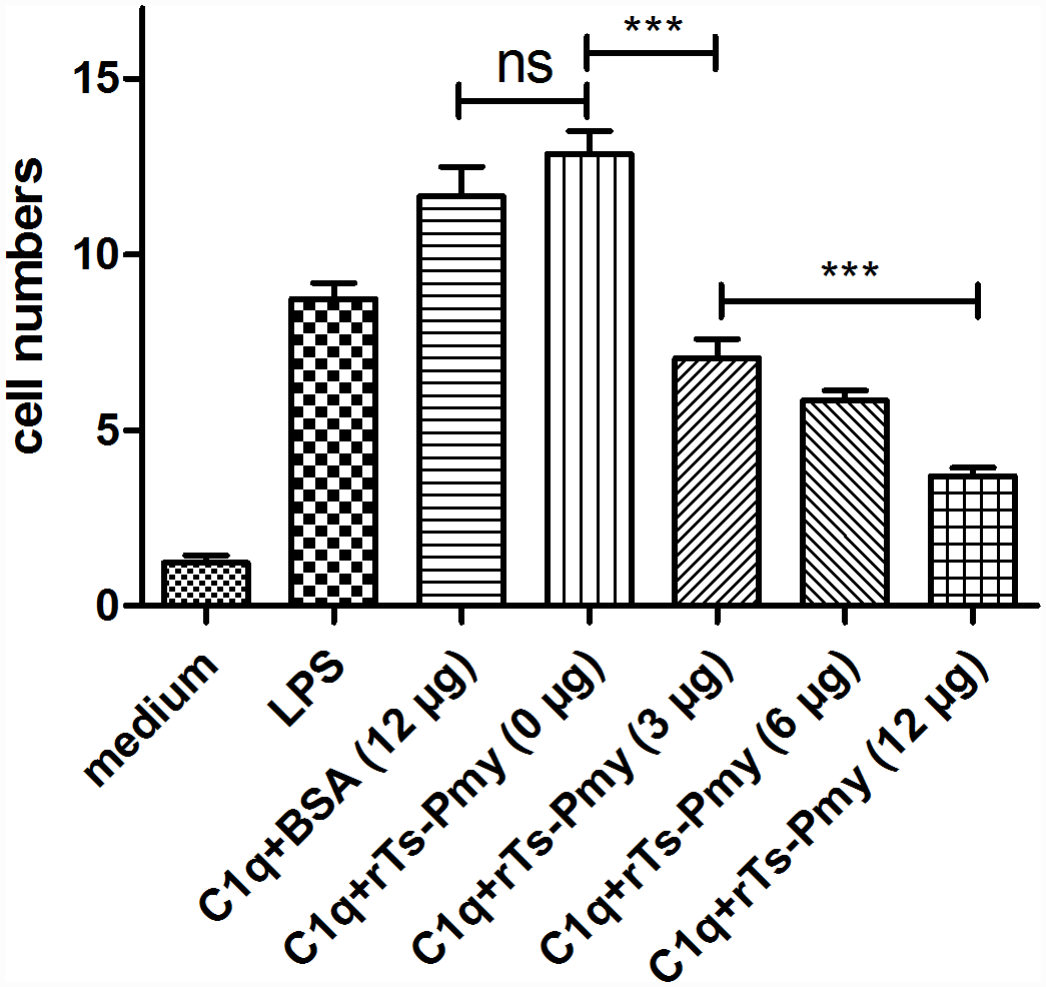
C1q-induced chemotaxis of THP-1-derived M2 macrophages was inhibited by r*Ts*-Pmy. Transwell-24-well plates were used for the migration assay. THP-1 cells were induced into M2 type macrophages in the upper chamber. LPS (100 ng/ml), C1q (10 nM) and C1q with different amounts of r*T*s-Pmy (0, 3, 6, or 12 μg) were added into the lower chamber. The cells that migrated though the membrane were counted under a microscope, and the cells from 8 randomly selected fields were counted. The results shown are means ± SD and are representative of three independent experiments. ***p<0.001. ns, no significant difference.

To investigate the effect of r*T*s-Pmy on C1q-induced chemotaxis of THP-1-derived macrophages, a migration assay using a transwell chamber was performed. Both human C1q and LPS significantly induced the migration of THP-1-derived M2 macrophages through the membrane (Fig 7). After incubating C1q with increasing amounts of r*T*s-Pmy (0, 3, 6, or 12 μg), the cell migration through the membrane was significantly reduced in a dose-dependent manner (***p<0.001). No obvious inhibition was detected in the group incubated with BSA at high concentration of 12 μg. The result revealed that r*T*s-Pmy inhibited the chemotaxis of M2 phenotype macrophages towards C1q.

## Discussion

Complement activation is regarded as the initial guardian for pathogen elimination. Due to the fundamental role of the complement system in immune defense, evading complement system attack is a crucial step for the survival of pathogens. Many studies have reported immune evasion strategies developed by pathogens targeting complement. For example, the *Staphylococcus* complement inhibitor (SCIN) inhibited complement C3 convertases, and *Pseudomonas* elastase (PaE) inhibited C3 in a proteolytic degradation-dependent manner [31]. Pathogens including bacteria, viruses and parasites seem to share similar strategies to escape the immune attack by complement. However, the mechanisms underlying the evasion from complement attack developed by *T*. *spiralis* were not well investigated.

Paramyosin is a structural muscle protein that is expressed only in invertebrates. In addition to forming thick myofilaments, paramyosin is also expressed on the surface of *S*. *mansoni* [12], *Echinococcus granulosus* [32] and *T*. *spiralis* [13]. Recent studies revealed that paramyosin expressed on the helminth surface might act as a potential immunomodulatory effector by targeting complement. *S*. *mansoni* paramyosin could inhibit complement activation and the immune response by binding to complement C8, C9 [12], C1q and IgG antibody [33]. *T*. *solium* paramyosin blocked the activation of C1 by binding to complement C1q [11]. In our previous study, we demonstrated that *T*. *spiralis* paramyosin was able to inhibit the formation of MAC by binding to C8 and C9 and therefore protected the parasites from attack by activated complement [13]. In this study, we demonstrated that *T*. *spiralis* paramyosin also targeted C1q, which is the initiator of the classical complement activation pathway.

C1 is the first component of the classical pathway and comprised of three subcomponents: C1q, C1r and C1s. C1q is a versatile pattern recognition molecule that can interact with different types of ligands and perform various biological responses in addition to the initiation of the classical complement pathway [34]. Both the complement and non-complement functions of C1q play a crucial role in the host immune response. In the present study, we demonstrated that *Ts*-Pmy (both the natural protein from adult worms and the recombinant protein) could bind to C1q, more specifically to the A chain of C1q, indicating that *Ts*-Pmy might interfere with classical complement activation. Subsequent results showed that C3 deposition onto classical pathway activator human IgM were reduced in the presence of r*Ts*-Pmy, confirming that the binding of r*Ts*-Pmy to C1q could inhibit the activation of the classical complement pathway indeed. Further investigation demonstrated that r*Ts*-Pmy inhibited the binding of C1q to IgM, suggesting that *Ts*-Pmy and IgM share the same binding site on the head region of C1q. *Ts*-Pmy’s binding on C1q blocks C1q’s binding to IgM or other immune complex, therefore inhibits the complement classical pathway activation. It may reflect one of the mechanisms that parasite-produced paramyosin inhibites the complement classical activation as a strategy to evade the complement-involved immune attack. The inhibition of C1q activation through binding to r*Ts*-Pmy may affect the final formation of MAC, which was directly reflected by reduced hemolysis compared to C1q without r*Ts*-Pmy. However, our previous study showed that *Ts*-Pmy also bound to C8/C9 except for C1q identified in this study. Therefore the *Ts*-Pmy induced inhibition of hemolysis may have resulted from the synergetic consequences of inhibiting both C1q and C8/C9 that reduce final MAC formation [13]. The results suggest that parasite-produced molecule(s) such as *Ts*-Pmy play roles in immunomodulating the host immune system by targeting a number of immune molecules and pathways.

In addition to complement which acts as the first line of innate immune defense, macrophages also play important roles in TH1- and TH2-mediated responses and eliminate pathogens directly or by associating with neutrophils and complement [35]. Macrophages or monocytes express complement receptor 1 (CR1) and other receptors on their surfaces [30,36,37] to interact with complement. It has been reported that C1q can not only directly bind to CR1 to activate macrophages, but also act as a chemokine to induce macrophage migration to inflammatory regions; therefore, C1q may play roles in the process of tissue damage and repair [27] and the elimination of the pathogen by phagocytosis [38]. In this study, we confirmed that C1q was able to bind to the surface of THP-1-derived M2-like macrophages, the addition of r*Ts*-Pmy reduced the binding of C1q on macrophages possibly through blocking C1q binding to the CR1 or other receptors on macrophages. The addition of r*Ts*-Pmy also reduced the C1q induced chemotaxis of THP-1-derived M2-like macrophages through a filter chamber, indicating that the binding of *Ts*-Pmy to C1q not only inhibited the C1q-initiated classical complement activation cascade but also impaired the C1q-induced migration of macrophages.

Together with our previous study, our results provide strong evidences that *T*. *spiralis* produces paramyosin as a potent immunomodulatory protein involved not only in the inhibition of complement activation through binding to C1q and C8/C9, but also in reducing the migration of macrophages to human C1q. Thus, paramyosin plays an important role in the defense against the host innate immune response and the survival of the parasite in the host, making it as a good preventive or therapeutic vaccine target against *Trichinella* infection. The C1q binding domain on r*Ts*-Pmy and how r*Ts*-Pmy inhibits the functions of C1q and other complement component(s) are under investigation.
